# Piezoelectric Outputs of Electrospun PVDF Web as Full-Textile Sensor at Different Mechanical Excitation Frequencies

**DOI:** 10.3390/polym16121728

**Published:** 2024-06-18

**Authors:** Fenye Meng, Jiyong Hu

**Affiliations:** 1 Fashion & Art Design School, Jiaxing Vocational & Technical College, Jiaxing 314036, China; mengfy@jxvtc.edu.cn; 2 Key Laboratory of Textile Science & Technology, Ministry of Education, Donghua University, Shanghai 201620, China

**Keywords:** piezoelectric outputs, PVDF web, mechanical excitation, frequency, electrospinning conditions

## Abstract

With the increasing application of electrospun PVDF webs in piezoelectric sensors and energy-harvesting devices, it is crucial to understand their responses under complex mechanical excitations. However, the dependence of the piezoelectric effect on mechanical excitation properties is not fully comprehended. This study aims to investigate the piezoelectric output of randomly oriented electrospun PVDF nanofiber webs fabricated through different electrospinning processes at various mechanical excitation frequencies. The electrospun PVDF web was sandwiched between two textile electrodes, and its piezoelectric output as a full-textile sensor was measured across a frequency range from 0.1 Hz to 10 Hz. The experimental results revealed that the piezoelectric output of the electrospun PVDF web exhibited a nearly linear increase at excitation frequencies below 1.0 Hz and then reached an almost constant value thereafter up to 10 Hz, which is different from the hybrid PVDF or its copolymer web. Furthermore, the dependency of the piezoelectric output on the excitation frequency was found to be influenced by the specific electrospinning process employed, which determined the crystalline structure of electrospun PVDF nanofibers. These findings suggest that determining an appropriate working frequency for randomly oriented electrospun PVDF nanofiber webs is essential before practical implementation, and the piezoelectric response mode in different mechanical activation frequency ranges can be used to detect different human physiological behaviors.

## 1. Introduction

A survey of the recent literature reveals the utilization of the piezo- and ferroelectric properties of electrospun PVDF (Piezoelectric Effect Polyvinylidene Fluoride) fibers for energy conversion, power generation, and various sensor applications [1,2,3,4,5,6]. Piezoelectric sensors made from electrospun PVDF fibers have been successfully employed to measure slowly varying signals, such as heart sounds, arterial pulse, human respiration, subtle muscle movements, and fall and voice recognition [7,8,9,10]. With the rapid development of wearable technology and smart medical devices, more and more works are focusing on the application of electrospun PVDF nanofibers/mats in detecting and measuring human behaviors and physiological activities [3,7,9]. Usually, analyzing the output voltage is considered the most crucial approach to evaluating the piezoelectric response of PVDF fibers used as sensors to record human physiological signals with a wide frequency range.

Besides the electrospinning process of PVDF nanofibers [10,11,12,13,14], the piezoelectric output is reported to be influenced by various factors, including the sample thickness and dimensions, the excitation method [15], and additives [16]. For these reasons, there exist variations in the output voltages reported by different researchers. Investigations have been conducted on the performance of piezoelectric devices with different fiber diameters and material thicknesses of electrospun PVDF webs [5,17,18,19]. It has been observed that an increase in mat thickness as well as in mat area size leads to a higher output voltage [20]. Furthermore, Persano et al. [21] found that under identical load conditions, fibers arranged in dense arrays exhibit a voltage output approximately two orders of magnitude higher than fibers in a random arrangement.

In addition to the geometrical dimensions of electrospun nanofibers, several studies have reported on the influence of the excitation method on piezoelectric efficiency. Fang et al. [22] observed the significant impact of frequency on the electrical output of randomly oriented electrospun PVDF nanofiber devices during repeated compressive impact–release cycles. Higher output signals were recorded with an increase in working frequency from 1.0 Hz to 10.0 Hz. They proposed that the frequency-dependent electrical output originates from the influence of the initial impact speed. However, at high impact frequencies, uneven output signals were observed due to the incomplete recovery of fibers from compressive deformation or mechanical vibrations from the pressing device itself. Furthermore, the duration of the occurrence of the output peak during recovery decreased as the impact frequency increased. Of course, they just observed the responses of PVDF nanofiber devices at 1.0 Hz, 5.0 Hz, and 10.0 Hz impact frequencies, and it is unknown whether the frequency-dependent electrical output always follows a consistent trend in the range from 1.0 to 10.0 Hz. Additionally, they did not measure the piezoelectric responses in PVDF nanofiber devices at activation frequencies below 1.0 Hz, where slow walking in rehabilitation training and normal human respiration occur.

Ren et al. [23] observed that the output voltage of P(VDF-TrFE) webs slightly increases with the frequency of the external force due to the dynamic response of the measurement circuit and the increasing accelerated speed of the force. They discovered that from 0.1 up to 20 Hz, the signals from the sensor based on P(VDF-TrFE; 77/23, molar ratios, concentrations of TrFE in the copolymer) maintain a one-to-one relationship with the external force. In comparison, the sensor based on P(VDF-TrFE; 55/45) can only withstand an external force up to 12 Hz and exhibits a constant line in a non-one-to-one relationship at 20 Hz. These phenomena indicate that piezoelectric responses are influenced by both mechanical excitation conditions and the fiber structure of the electrospun web. Although they observed the frequency-dependent electrical output, it is still unclear how the peak-to-peak response changes with the external force frequency.

Lee and Ahn et al. [24] developed a novel hybrid sensor by stacking PVDF and thermoplastic polyurethane electrospun nanofiber webs, enabling the simultaneous measurement of static and dynamic pressure across all frequency ranges. The experimental results demonstrated a gradual increase in output voltage with the excitation frequency, as is consistent with Fang’s results [22]. Zeng et al. [25] utilized a PVDF-NaNbO_3_ nanofiber nonwoven fabric as an active piezoelectric component, with elastic conducting knitted fabric serving as the top and bottom electrodes. Their findings demonstrated that the output gradually increased in response to a dynamic impact force ranging from 1 Hz to 5 Hz. Furthermore, it was observed that the electric output was significantly influenced by both the frequency and magnitude of the applied excitation force. Li et al. [26] fabricated a PVDF nanofiber membrane sandwiched between two PVDF-rGO electrode membranes, where the output exhibited nearly linear growth with increasing impact frequency from 1 to 30 Hz. Moreover, at higher impact forces ranging from 5 N to 36 N, there was an accelerated rate of increase in the output with the frequency; however, overall, there existed a linear relationship between the output and excitation force.

On the contrary, Wang et al. [27] conducted an investigation on the properties of an electrospun PVDF mat as a sensor under forces with frequencies of 0.05, 0.1, 1, 5, 15, and 20 Hz. It was observed that at frequencies below 5 Hz, the peak responses corresponding to the application and release of square-wave loads increased while maintaining a relatively stable level as the excitation frequency increased. Li et al. [28] showed a decreasing piezoelectric output of electrospun core–sheath PVDF piezoelectric fibers in the force frequency range from 1.0 Hz to 5.0 Hz, where the core was stainless steel fibers and the sheath was PVDF, and the piezoelectric output first increased with the force frequency up to 3.0 Hz and then decreased. Additionally, in the case of melt-spun piezoelectric bicomponent fibers consisting of PVDF as the sheath component and a conductive composite comprising carbon black (CB) and high-density polyethylene (HDPE) as the core component, it was found that the piezoelectric output exhibited a rapid increase with sinusoidal oscillating frequencies ranging from 0.1 Hz to 1.0 Hz before reaching nearly constant values [29].

Previous research has observed variations in piezoelectric output under dynamic force excitation with different frequencies and intensities. However, the dependence of the piezoelectric output on the excitation frequency and electrospinning processes remains unknown, and it will limit the choice of electrospun PVDF devices in practice and the performance comparison among these devices. Also, most previous works used copper foil as the electrode in their tests, and it may not show the mechanical activation response of electrospun PVDF webs in the context of wear. Therefore, this study aims to investigate the piezoelectric response capabilities of electrospun PVDF webs with different electrospinning parameters influencing jet formation (applied voltage, flow rate, and needle diameter) at various mechanical excitation frequencies, as well as a full-textile packaging ensemble, and explain the underlying mechanism. The results will serve as a guideline for designing suitable electrospun PVDF webs for wearable applications such as full-textile sensors or human-motion-energy-harvesting devices.

## 2. Preparation and Testing

### 2.1. Preparation for PVDF Fibrous Mats

To achieve PVDF nanofiber mats with a high piezoelectric effect, precise control over the fabrication conditions is necessary. A customized electrospinning setup described in previous work [30,31] was used to prepare the PVDF nanofibrous mats. In short, PVDF pellets (MW 172,000, Sigma-Aldrich, Shanghai, China) were dissolved in a DMF-Acetone solvent mixture (4/6 *v*/*v*) to obtain 10 wt% PVDF solutions, and then the solutions were placed in the syringe of the electrospinning setup. After that, nanofibrous mats with different properties were prepared by changing the electrospinning processing parameters, including the applied voltage across two electrodes, the feeding solution flow rate in milliliter per hour, and the syringe needle tip diameter. Here, the range of electrospinning parameters is based on published work on non-bead uniform nanofibers, where the beads in the electrospinning PVDF nanofiber significantly degraded the piezoelectric response [32]. Also, the time for collecting the nanofibers during electrospinning was controlled to obtain a statistically uniform nanofiber mat with the same thickness due to the dependence of the piezoelectric output on the thickness of samples [17].

Based on previous research [30], three sets of experiments and fifteen runs were conducted, as outlined in Table 1. The electrospinning parameters, including the high applied voltage, feeding flow rate, and needle tip diameter, were varied to investigate their impact on the piezoelectric output of PVDF nanofibrous webs as sensors at different excitation frequencies.

### 2.2. Piezoelectric Device and Measuring Methods

**Figure 1:**
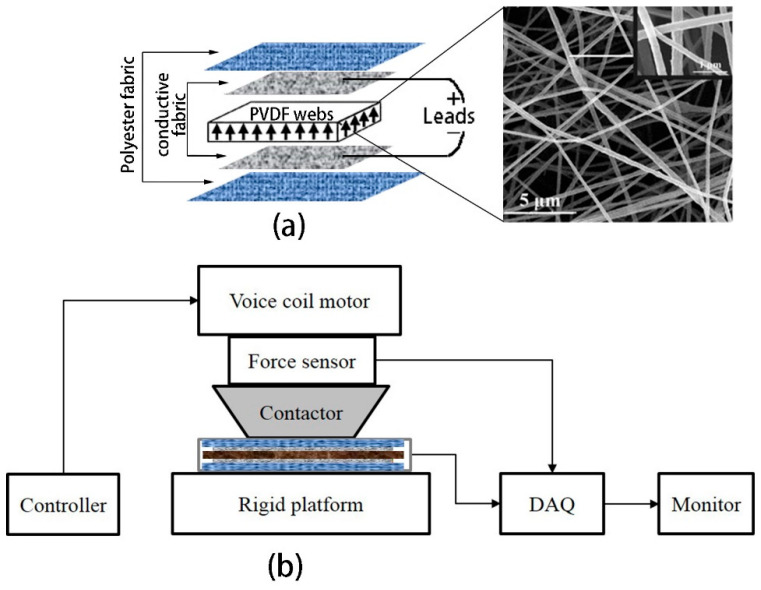
(**a**) A schematic representation of the full-textile pressure sensor and (**b**) a schematic diagram of the self-made experimental setup for piezoelectric characterization.

In order to detect the piezoelectric output from the electrospun PVDF nanofiber web, a fully integrated textile pressure sensor was fabricated, as depicted in Figure 1 (top left). The bottom electrode, consisting of a square piece of silver-plated polyester fabric measuring 3 × 3 cm with a thickness of 47 ± 10 μm and a surface resistance of 910 ± 53 Ω/cm^2^, was securely attached to the outer surface of the nanofiber web. A top electrode made of silver-plated polyester fabric, which had been used to wrap the collector during electrospinning, was employed. Finally, the entire sandwich structure (top electrode–nanowebs–bottom electrode) was encapsulated using one-sided adhesive electrically insulated cloth with a thickness of approximately 100 μm. The sensor prototype exhibits excellent insulation properties due to its larger web area compared to that of the upper and lower electrodes. The simplified equivalent circuit diagram for detecting the piezoelectric signal is illustrated in Figure 1b. Silver-plated polyester threads (400D/96F, 12 Ω/m) serve as leads for connecting both ends of the piezoelectric sensor prototype to a signal acquisition device (S-2400, Keithley, Cleveland, OH, USA), which possesses an infinitely large input impedance (>10 kMΩ), enabling the measurement of the open-circuit voltage.

The as-fabricated prototype of the full-textile PVDF sensor was characterized using a customized cyclic compression setup. This setup provided a series of dynamic excitation force pulses with controlled pulse pressures and a frequency ranging from 0.3 to 10 Hz, as shown in Figure 1. A rigid contactor with a flat circular tip measuring 2.0 cm in diameter was designed for this purpose. A preload of 10 mN was applied to the top of the PVDF sensor prototype before directly applying a maximum magnitude of 15 N during the measurement process. The piezoelectric output and applied force were simultaneously acquired, with the peak voltage being sampled and each listed value representing an average value obtained from 20 peaks.

## 3. Results

**Figure 2:**
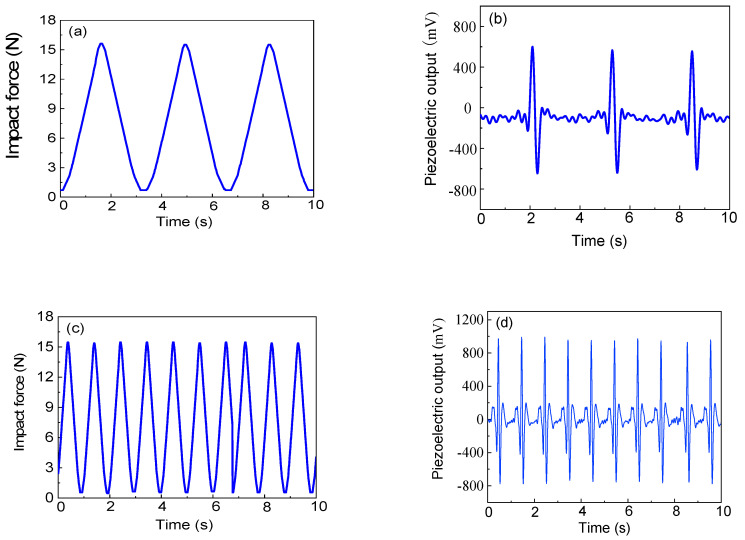
The real-time force and piezoelectric output of the packaged full-textile sensor prototype at different mechanical excitation frequencies: (**a**) 0.3 Hz; (**b**) 1.0 Hz. The sensitive material of the sensor prototype was obtained at an applied voltage of 20 kV; (**c**) the time-dependent activation force; (**d**) the piezoelectric output under the dynamic activation force.

The piezoelectric output of the electrospun PVDF web under mechanical excitation forces with different frequencies at an applied electrospinning voltage of 20 kV is presented in Figure 2. The results demonstrate a consistent response and excellent repeatability. Additionally, it is observed that the piezoelectric output is influenced by the excitation frequency. At an excitation frequency of 0.3 Hz, the average piezoelectric output of the packaged PVDF web measures 565 mV, whereas at an excitation frequency of 1.0 Hz, it increases to 946 mV. Evidently, the piezoelectric output of the electrospun PVDF web can be affected by variations in excitation frequency.

**Figure 3:**
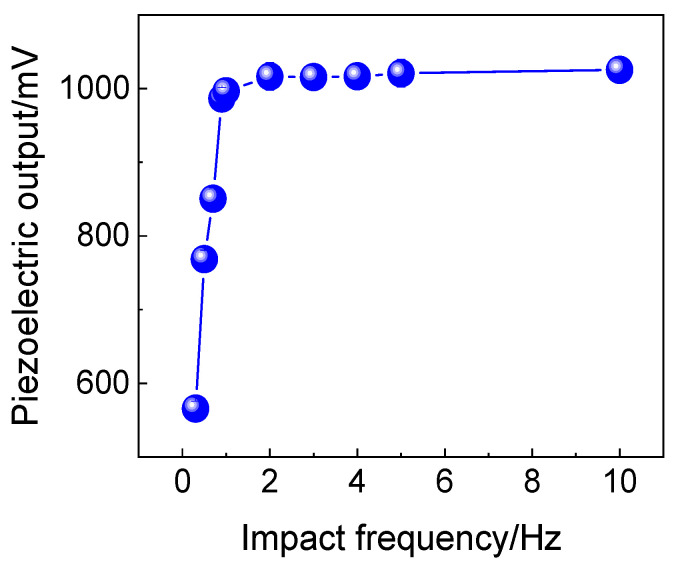
The dependence of piezoelectric output on the mechanical excitation frequency. The full-textile sensor prototype is the same as that in Figure 2.

The piezoelectric outputs of the sensor prototype at different mechanical excitation frequencies were extracted. The dependence of the piezoelectric output of the sensor prototype in Figure 2 on the mechanical excitation frequency is illustrated in Figure 3. It can be observed that at frequencies below 1.0 Hz, there is a significant increase in piezoelectric output from 565 to 993 mV, followed by a relatively stable level up to an excitation frequency of 10 Hz. This phenomenon is consistent with Nilsson’s work [29]. Also, a similar result was observed by Wang [27], where the piezoelectric output has a slow increase with the force frequency from 0.1 up to 5.0 Hz and remains at a constant level at 10.0 and 20.0 Hz forces. The different observed increase rates of piezoelectric output with the activation frequency may come from the electrospinning conditions as well as the packaging materials of the PVDF device as a sensor, as will be discussed in the next section. On the other hand, from the results reported by Ren et al. [23], the output voltage slightly increases with the frequency of force. In fact, in Figures 9 and 10 in [23], the peak-to-peak piezoelectric output of the P(VDF-TrFE; 77/23) nanofiber mats remain at nearly the same level with the increasing mechanical frequency from 10 to 20 Hz, and that of P(VDF-TrFE; 45/55) nanofiber mats is at nearly same level with frequencies from 1.0 up to 10.0 Hz and even remains near the zero level at 20.0 Hz. Notably, most previous work related to PVDF nanofiber mats with additives showed increasing piezoelectric outputs with mechanical excitation frequencies above 1.0 Hz, and the additives probably change the crystalline structure of the PVDF component of electrospun nanofibers. Generally, this work systematically uncovered the relationship between the piezoelectric output and the mechanical activation frequency.

In theory, the piezoelectric output of the sensor prototype is a dynamic mechanical excitation loading process involving charging and discharging. Consequently, the piezoelectric output is influenced by the electrical properties of the sensor prototype. The electrical model of the piezoelectric sensor resembles a high-pass filter, implying that at high frequencies, the piezo signal remains unaffected, while it is attenuated below a specific frequency, known as the cut-off frequency (fc). Previous observations have indicated that PVDF nanofiber webs exhibit high electrical resistance and low electrical capacity [10]. As a result, storing charge released by the PVDF web during low-frequency mechanical excitation becomes challenging, with even a portion of the piezoelectric charge output being occupied by its high internal electrical resistance. At higher frequencies where excitation periods may be shorter than the relaxation time for the full-textile-packaged electrospun PVDF nanofiber web, losses in charge due to internal electrical resistance are minimal, and thus, the excitation frequency has little impact on the piezoelectric output. Therefore, it is imperative to investigate how electrospinning conditions affect the relationship between the frequency and piezoelectric output of full-textile-packaged electrospun PVDF nanofiber webs.

## 4. Discussion

**Figure 4:**
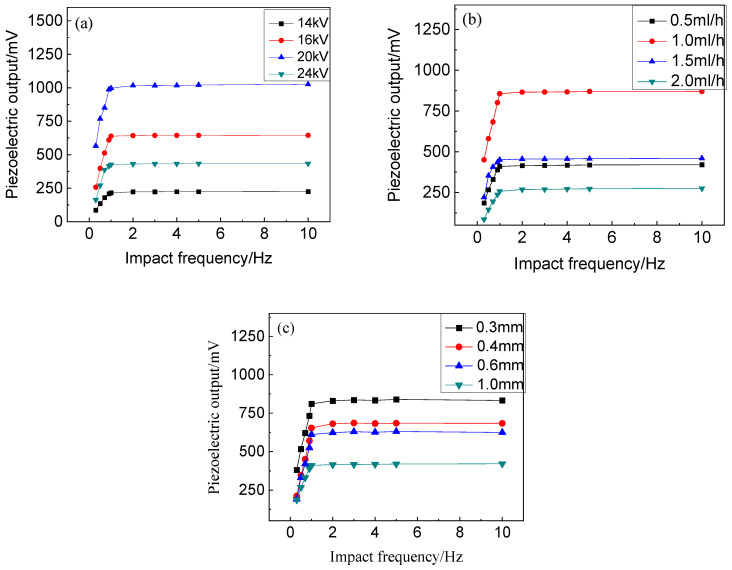
The effect of the excitation frequency on the output of PVDF nanofiber webs under different electrospinning conditions as full-textile sensors. (**a**) applied voltage; (**b**) feeding flow rates; (**c**) needle-tip diameter.

To investigate the intrinsic mechanics underlying the dependence of the piezoelectric output on the excitation frequency, we conducted tests on the piezoelectric outputs of electrospun PVDF webs under various electrospinning conditions, as depicted in Figure 4. The variation in the piezoelectric output with the mechanical excitation frequency is closely associated with the specific electrospinning conditions employed. For each set of electrospun PVDF webs produced under different electrospinning conditions, the piezoelectric output exhibits a rapid increase up to approximately 1 Hz and then reaches a near-constant level. Moreover, the rate at which the piezoelectric output, as well as the stable level, increases at low excitation frequencies is positively correlated with a higher applied voltage and feeding flow rate. At higher excitation frequencies, there exists a positive relationship between the spinning parameters and piezoelectric output. Fundamentally, distinct electrospinning conditions lead to variations in the crystalline structure [11,12], which determine the fraction of the β crystalline component responsible for the observed piezoelectric effect. Additionally, these conditions also result in differences in nanofiber dimensions that enhance the overall piezoelectric responses through electromechanical interactions among adjacent fibers—a cooperative effect dependent on specific geometrical parameters [21]. In this regard, it is worth noting that it is primarily the morphology of electrospun PVDF nanofibers, determined by their respective electrospinning conditions, that governs their performance across different excitation frequencies. In this sense, the difference in piezoelectric output levels between our work and Wang’s result [27] partly comes from different electrospinning conditions. Of course, the electrode or packaging materials in this work are different from those in most previous work, where copper foil, conductive knitted fabric, or PVDF-rGO membranes were used as electrodes [23,24,25,26]. The different compression recovery properties of these electrode materials may affect the mechanical deformation of the laminated structure of a piezoelectric device, so a different change in the piezoelectric response with the mechanical activation frequency was observed. In this sense, the total effect of the PVDF nanofiber web, as well as the packaging and electrode textiles, determines the dynamic frequency of the packaged piezoelectric sensor device.

Generally, across various electrospinning conditions, the PVDF sensor prototype consistently exhibited a discernible correlation with the mechanical excitation frequency. Specifically, at low excitation frequencies, the piezoelectric output experienced a significant surge, while at high excitation frequencies, it either gradually increased or remained stable. Consequently, under these specific electrospinning conditions, full-textile sensors composed of PVDF nanofiber webs are well suited for monitoring dynamic forces exceeding 1.0 Hz. In this regard, it is important to note that this particular type of PVDF sensor prototype possesses its own resonance frequency, which necessitates appropriate filtering measures. The application schematic effectively demonstrates the impact of employing a high-pass filter on the frequency response. On the other side, the nearly linear change in the piezoelectric output with the mechanical excitation frequency below 1.0 Hz can be used to discriminate breathing and slow-walking behaviors among a population of subjects [33].

## 5. Conclusions

This study systematically investigated the piezoelectric output of electrospun PVDF nanofibrous webs as full-textile sensors using varying mechanical excitation frequencies and electrospinning process parameters. The experimental results revealed a significant correlation between the piezoelectric output of electrospun PVDF nanofibrous webs and the mechanical excitation frequency. Specifically, the piezoelectric output increased dramatically at frequencies below 1.0 Hz before nearly stabilizing, and the effect of the excitation frequency could be adjusted by controlling electrospinning process parameters as well as additives. These are not previously observed phenomena for hybrid PVDF or PVDF copolymer webs with additives. Also, the results indicate that the correlation between the piezoelectric output and the mechanical excitation frequency mainly depends on the crystalline structure, not on the packaging or electrode materials. It was concluded that sensors made from pure PVDF nanofibrous webs with electrospinning technology are best suited for monitoring dynamic forces with changing frequencies above 1.0 Hz and are suitable for the discrimination of low-frequency activities, such as breathing, among a population of human subjects. These findings highlight the importance of considering the operating frequency range and sensitivity when using pure electrospun PVDF nanofibrous webs as piezoelectric sensors.

## Figures and Tables

**Table 1:** Experimental designs with controlled electrospinning parameters.

Applied Voltage (kV)	Flow Rate (mL/h)	Needle Tip Diameter (mm)
14, 16, 18, 20, 22, 24	1.0	0.6
18	0.5, 1.0, 1.5, 2.0, 3.0	0.6
18	1.0	0.3, 0.4, 0.6, 1.0

## Data Availability

Data are contained within the article. The original contributions presented in the study are included in the article. Further inquiries can be directed to the corresponding author.
